# Ixodid and Argasid Tick Species and West Nile Virus

**DOI:** 10.3201/eid1004.030517

**Published:** 2004-04

**Authors:** Charles Henderson Lawrie, Nathalie Yumari Uzcátegui, Ernest Andrew Gould, Patricia Anne Nuttall

**Affiliations:** * Centre for Ecology & Hydrology, Oxford, United Kingdom

**Keywords:** West Nile virus, virus, ticks, Ixodidae,Argasidae,transmission,co-feedingtransmission.

## Abstract

Control of West Nile virus (WNV) can only be effective if the vectors and reservoirs of the virus are identified and controlled. Although mosquitoes are the primary vectors, WNV has repeatedly been isolated from ticks. Therefore tick-borne transmission studies were performed with an ixodid (*Ixodes ricinus*) and an argasid tick species (*Ornithodoros moubata*). Both species became infected after feeding upon viremic hosts, but *I. ricinus* ticks were unable to maintain the virus. In contrast, *O. moubata* ticks were infected for at least 132 days, and the infection was maintained through molting and a second bloodmeal. Infected *O. moubata* ticks transmitted the virus to rodent hosts, albeit at a low level. Moreover, the virus was nonsystemically transmitted between infected and uninfected *O. moubata* ticks co-fed upon uninfected hosts. Although ticks are unlikely to play a major role in WNV transmission, our findings suggest that some species have the potential to act as reservoirs for the virus.

The first report of a West Nile virus (WNV) outbreak within the Western Hemisphere occurred in 1999 in New York City and resulted in human, equine, and avian deaths (*1*). Since 1999, WNV has been found in an additional 44 states of the United States as well as in parts of Canada, the Caribbean, and South America (*2*,*3*). During 2002 more than 4,000 people diagnosed with WNV and 284 deaths were reported in the United States (latest records available from: http://www.cdc.gov/ncidod/dvbid/westnile/index.htm).

WNV is a member of the genus *Flavivirus* that contains over 70 identified viruses. Most of these viruses are vectored by mosquitoes or ticks, although a few have no known vectors (*4*). WNV has been isolated from 43 species of mosquito in the United States (*5*), the most important of which is *Culex pipiens* (*6*). It has also been isolated from hard (ixodid) and soft (argasid) tick species in regions of Europe, Africa, and Asia (*7*–*13*) where WNV is endemic. Ticks rank second only to mosquitoes in their importance as vectors of human pathogens and transmit a greater variety of infectious agents than any other arthropod group (*14*). However, whether or not ticks are major vectors of WNV has not been adequately investigated.

Current strategies to control WNV in the United States are largely based on measures to avoid exposure and to control vector species, but at present only mosquito species are targeted by government surveillance and preventive control programs (*15*). Resident U.S. tick populations could also play a role in the WNV transmission cycle in the current outbreak. We investigated an argasid tick species and an ixodid tick species for their competence as vectors and reservoirs of the New York strain (NY99) of WNV.

## Materials and Methods

### Ticks

We tested a hard tick species, *Ixodes ricinus*, and a soft tick species, *Ornithodoros moubata*, for their vector competence with WNV (NY99 strain). These species are not native to the United States and were chosen mainly for their availability. *O. moubata* ticks were considered potential vectors for the Eg101 strain of WNV in a study by Whitman and Aitken in 1960 (*16*). *I. ricinus* ticks are the primary vectors of *Borrelia burgdorferi*, the agent causing Lyme disease in Europe and are important vectors of the flaviviruses tick-borne encephalitis virus (TBEV) and Louping-ill virus (LIV) (*17*).

Ticks were taken from colonies reared and maintained for many generations at the Centre for Ecology and Hydrology Oxford according to standard methods (*18*). Colony ticks were WNV negative by reverse transcriptase–polymerase chain reaction (RT-PCR) testing (15 members of each species tested).

### Virus and Viral Assays

The WNV strain used (NY99) was supplied by Robert Shope, University of Texas. High-titer mouse brain suspension stocks of WNV (2.9 x 10^7^ PFU/mL^-1^) were diluted in phosphate-buffered saline (PBS) to a concentration of 10^5^ PFU/mL^-1^ before use. Viral stocks and the serum samples from infected mice were tested for infectious virus by plaque assays on pig kidney epithelial cells as described previously (*19*), by using a 3% carboxymethylcellulose overlay.

### Tick Infection and Co-feeding Transmission Experiments

Seven groups of six BALB/c mice (female, 4–6 weeks old) were injected subcutaneously with 10^4^ PFU of WNV. Three of the mice were bled daily from the tail to follow the course of viremia by plaque assay. Two groups of mice were infested with *I. ricinus* nymphs (20 per mouse); one group was infested 3 days before infection, the other 4 days after infection. The other five groups of mice were infested with second instar *O. moubata* ticks (10 per mouse) on either the same day (day 0) or 1, 2, 3, or 4 days after infection. After the initial experiment, and to increase the number of positive ticks available for experimentation, 12 additional mice were infested with *O. moubata* 2 days after infection with WNV.

Ticks housed in gauze-covered neoprene feeding chambers on mice (*18*) were removed when fully engorged, 24 hours after infestation in the case of *O. moubata* ticks and 6 days after infestation in the case of *I. ricinus* nymphs. The engorged ticks were stored at 20°C in KCl-saturated desiccators until testing for WNV or until ready for a further bloodmeal, as indicated in Table 1. After storage, the ticks (pools and individual ticks) were homogenized in 500 μL of PBS by using plastic homogenizers under sterile conditions. The homogenates were frozen and stored at –70°C until analyzed. Tick homogenates were assayed for infectious virus antigen (by immunofluorescence assay) and viral RNA (by RT-PCR) as shown in Table 1.

**Table 1:** Results of immunofluorescence assay (IFA) or nested reverse transcriptase–polymerase chain reaction (RT-PCR) from *Ornithodoros moubata* and *Ixodes ricinus* ticks fed on West Nile virus–inoculated BALB/c mice or noninfected mice (co-fed ticks)

Species	Developmental stage	Days from infection to infestation	Days after engorgement^a^	IFA^b^ +/–	RT-PCR^c^ (no. positive/no. tested)	
*O. moubata*		0	1, 2, 7	– (8)	ND	
First bloodmeal (infected mice)	Second instar	1	1–7	– (5)	ND	
		2	1–7, 14	+ (5)	+ (5)	
		3	1–7, 14	+ (5)	+ (5)	
		4	1, 3, 7	– (5)	ND	
	Third instar	2	22	+ (5)	ND	
		3	22	+ (5)	ND	
		2	132	+ (5)	7/14	
Second bloodmeal (uninfected mice)	Third instar	2	60 (3)	+ (5)	+ (5)	
		2	64 (7)	+ (5)	+ (5)	
	Fourth instar	2	75 (25)	+ (5)	+ (5)	
Uninfected co-fed *O. moubata* ticks	Second instar	N/A	5	ND	15/66	
	Third instar	N/A	45	ND	4/15	
*I. ricinus* First bloodmeal (infected mice)	Nymph	4	2	ND	0/12	
		–3	2	ND	2/12	
		–3	30	ND	0/25	
BALB/c mice^d^	N/A	N/A	N/A	– (1)	1/17	

^a^Number of days after the ticks had completed feeding on inoculated mice when ticks were tested for virus infection. Where given, parentheses depict ticks that had fed a second time and the number of days after which the ticks were tested. ^b^Tick homogenate samples were scored positive if >10% of inoculated C6/36 cells showed specific fluorescence with both 813 and 546 monoclonal antibodies. Numbers of ticks in each pool are shown in parentheses. ^c^Where indicated by +, pools of ticks were tested; numbers of ticks in each pool are shown in parentheses. ND, not done. ^d^Mice were infested with infected *O. moubata* ticks and after 14 days were sacrificed and the brain homogenates tested by IFA and RT-PCR. N/A, not applicable.

Co-feeding transmission experiments were carried out by infesting clean BALB/c mice (n = 7, Harlan, UK) with 10 third instar *O. moubata* ticks 57 days after they had taken an infectious bloodmeal, and 10 uninfected ticks (second instar) in separate feeding chambers. The two feeding chambers were separated by at least 1 cm.

To investigate tick-to-host transmission, 10 BALB/c mice were infested with cohorts of 5, 10, 15, or 20 third instar *O. moubata* ticks 57 days after an infectious bloodmeal. Fifteen days after infestation, the mice (including those used for co-feeding) were euthanized; brains were removed, homogenized in 1 mL of sterile PBS, and stored at –70°C until they were tested for WNV.

### Immunofluorescence Assay

Samples of tick (or mouse brain) homogenate (100 μL) were used to infect 2 x 10^6^ C6/36 mosquito cells in a total of 3 mL L-15 medium containing 7% fetal calf serum (Gibco-BRL, Paisley, UK) in 30 mm Petri dishes that contained glass coverslips. Infected cells were incubated at 28°C for 3 days. Cells were fixed in cold acetone and treated according to standard methods (*19*). Infected cells were fluorescein isothiocyanate–labelled with a broadly reactive flavivirus E-protein monoclonal antibody (MAb 813) (*20*) or a WNV-specific monoclonal antibody (MAb 546) (*21*). Labelled cells were visualized by using an Olympus epifluorescence microscope. Uninfected cells were used as negative controls and cells infected with the original viral stock as positive controls. Tick samples were deemed positive when more than 10% of the cells showed specific fluorescence with both monoclonal antibodies.

### Nested RT-PCR Assay

RNA was extracted from homogenized samples (100 μL) by using RNAgents total RNA extraction kit in accordance with the manufacturer’s instructions (Promega, Madison, WI). cDNA synthesis was carried out with Superscript II reverse transcriptase (Invitrogen, San Diego, CA) and 3′(1) primer (Table 2) for 50 min at 42°C, in a total volume of 20 μL. PCR was carried out on the cDNA (1 μL) by using 5′(1) and 3′(1) primers. Nested PCR was carried out on 1 μL of the first-round PCR product using the nested primers 5′(2) and 3′(2). All PCR reactions were carried out in a 50-μL volume with REDTaq DNA polymerase (Sigma Chemical Co., St. Louis, MO). A Hybaid Touchdown thermal cycler was used with the following program: 94.5°C for 1 min, 30 cycles of 94°C for 40 s, 56°C for 1 min, and 72°C for 1 min, followed by a final extension step of 72°C for 10 min. Viral stock, RNA extracted from uninfected ticks, and PBS-only samples were used as control reactions. Positive samples gave a PCR product of approximately 1.2 kbp. This method could detect RNA from a viral stock equivalent of 9 PFU (data not shown).

**Table 2:** Nucleotide sequences of primers used in first round (5′[1] and 3′[2]) and second round (5′[2] and 3′[2]) of nested RT-PCR^a^

Primer	Sequence	Position in WNV (NY99)^b^
5′(1)	CCATATGAATTCCATGAGTGCTATCAATCGGCGGAG	31 aa upstream (C gene) from start of PrM gene (373)
3′(1)	CATATGCGGCCGCTTACTAGGTGATTGATCTGTTGTTCTCC	31 aa downstream (NS1) from end of E gene (2,562)
5′(2)	CATATGCGGCCGCTTACTACCGGTCCATCCAAGCCTC	Start of E gene (967)
3′(2)	CCATATAGATCTCGGAGGTCATTCAACTGCCTTGGAATGAGC	395 aa into E gene (2,152)

^a^RT-PCR, reverse transcriptase–polymerase chain reaction; aa, amino acids. ^b^Nucleotide position in relation to complete genome sequence of WNV (NY99) shown in parentheses (accession no. AF19685).

To confirm the identity of RT-PCR products, PCR products were gel purified with QIAquick (Qiagen, Crawley, UK) columns in accordance with manufacturer’s instructions. The purified DNA was sequenced with an ABI automatic sequencer and the nested primers 5′(2) and 3′(1) and a primer based on the internal sequence of the E gene of WNV (not shown).

## Results

### Host-to-Tick Transmission

BALB/c mice infected with WNV were weakly viremic 2 and 3 days after injection, with mean titers of 6 x 10^3^ and 3 x 10^3^ PFU/mL^-1^ blood respectively. After 4 days, viremia was no longer detectable by plaque assay, although severe neurologic disease developed in the mice after 5 or 6 days, and they were euthanized. *O. moubata* ticks that had fed on mice on days corresponding to the viremic period (i.e., days 2 and 3 after infection), but not those fed outside this period, contained viral antigen as measured by immunofluorescence assay (IFA) (Table 1). Two days after engorgement, 17% (n = 12) *I. ricinus* ticks that started to feed on hosts 3 days before WNV injection, but not those that had started to feed 4 days after injection, were positive for WNV RNA. When the former group of ticks was tested 28 days later, no evidence of infection was found. Infected *O. moubata* ticks, in contrast, maintained the virus after molting into the next instar (i.e., third instar); following a second, noninfectious bloodmeal; and after molting a second time into fourth instars. Fifty percent of the individual ticks (n = 14) tested by RT-PCR were positive for WNV RNA when examined 132 days after the initial infectious bloodmeal.

### Co-feeding Transmission

Five days after engorgement, 23% (n = 66) of uninfected second instar *O. moubata* ticks that had co-fed with infected cohorts of third instar ticks on noninfected mice were positive for WNV RNA (Table 1). The remaining unfed ticks (n = 15) were tested after they had molted into third instars, 45 days after co-feeding. Four of these ticks (26%) were positive for WNV RNA. The identities of the PCR products obtained from three positive samples were confirmed by sequence analysis.

### Tick-to-Host Transmission

Infected cohorts of *O. moubata* ticks (third instar) were fed on uninfected mice to investigate tick-to-host transmission. Of the 17 uninfected mice used (including mice used in co-feeding experiments), none showed clinical signs of infection. One of the brains tested, from a mouse infested with an infected cohort of 20 ticks, was positive by RT-PCR but negative when tested by IFA (Table 1). The PCR product was sequenced to confirm the identity of WNV.

## Discussion

Laboratory studies from the 1950s suggested that some tick species might serve as competent vectors for WNV. Hurlbut and Taylor (1956) showed that *O. savignyi* and *O. erraticus* ticks were infected after feeding on mice inoculated with the Ar-248 strain of WNV, but transmission from infected ticks to mice was not observed (*22**,**23*). Vermeil et al. (1959) infected *O. maritimus* and *O. erraticus* ticks by feeding on inoculated (Uganda 28B strain) chickens, guinea-pigs, mice, or gerbils. Infected ticks transmitted the virus to uninfected mice (*24*). More recently, an artificial membrane system was used to infect *Argas arboreus* ticks, which were then able to transmit the virus to uninfected hosts, although transstadial transmission of WNV was not observed (*25*,*26*).

Our study demonstrated that both *I. ricinus* and *O. moubata* ticks become infected with WNV (NY99 strain) through feeding on virus-infected rodent hosts, but only when these hosts were viremic (i.e., systemic transmission). Thirty days after engorgement, we no longer found any evidence of WNV infection in the *I. ricinus* ticks. This finding suggests that nymphs of this tick species do not support replication of the virus, and therefore are not competent vectors for WNV. By extrapolation, the closely related tick species, *I. scapularis* (the main U.S. Lyme disease vector) is also unlikely to be a competent vector of WNV, although this hypothesis will need to be confirmed experimentally.

In contrast, infected *O. moubata* ticks maintained infectious virus for at least 132 days (length of experiment), and WNV persisted transstadially through at least two developmental stages. Evidence for tick-to-host transmission of WNV was found in our study, although the level of infection observed (subclinical) makes assessing its importance without further investigation difficult. Whitman and Aitken (1960) observed much higher levels of transmission from WNV-infected (Eg101 strain) *O. moubata* ticks to day-old chicks but only when very high feeding densities were used (an average of 49 ticks per chick) (*16*). Although ticks often feed in large numbers on individual hosts (*27*), tick-to-host transmission appears to be very inefficient when compared to mosquito transmission of WNV (*23*). Consequently, this mode of transmission is unlikely to be important in the natural transmission cycle of WNV. Perhaps higher levels of infection (and therefore transmission) would be found with ticks that feed on birds, the natural reservoir hosts of WNV. Some avian species exhibit much higher (>10^10^ PFU/mL serum) and more prolonged viremia when infected with WNV than the mice used for this investigation (*28*,*29*). Although neither of the tick species that we tested are obligate bird feeders, *I. ricinus* ticks often feed on pheasants in the United Kingdom (*30*), and several species of *Ornithodoros* ticks feed almost exclusively on birds, for example, the *O. capensis* group of ticks that are established along the southern coast of the United States (*31*). As members of this group have been shown to be competent vectors for WNV (*24*), these ticks could represent a reservoir of the virus in the United States.

The transmission of flaviviruses such as TBEV and LIV from infected to noninfected ixodid ticks through co-feeding on nonviremic hosts (nonsystemic transmission) is a well-established phenomenon (*32*). Indeed, this mode of transmission is believed to play a substantial role in the epidemiology of these diseases (*27*). We tested for co-feeding transmission of WNV between infected and uninfected *O. moubata* ticks. More than 22% of the uninfected ticks were positive for WNV RNA 5 days after co-feeding. A similar percentage of ticks were positive 40 days later, after having molted to the next developmental stage. As co-fed ticks were in contact with the mice for <24 hours, this finding strongly suggests that WNV was nonsystemically transmitted between infected and uninfected ticks, since viremia had insufficient time to develop. Our study represents the first unequivocal report of co-feeding transmission by an argasid tick species. Argasid ticks, unlike ixodid ticks, typically feed for <2 hours. Vesicular stomatitis virus has been transmitted between infected and noninfected co-feeding black flies (*Simulium vittatum*), insects that typically feed for 4–5 min (*33*). Langerhans cells are believed to be the agents of viral transmission between feeding sites of infected and noninfected co-feeding hard ticks (*32*,*34*). Langerhans cells, which are susceptible to WNV infection (*35*), have been shown to migrate rapidly (within 2 hours) from localized antigen-stimulated epidermal sites (*36*). Therefore, these cells could possibly play a similar role in the co-feeding transmission of WNV by soft tick species.

Although this study is not exhaustive, it does demonstrate that tick species can become infected with the U.S. strain of WNV through feeding upon infected hosts and through co-feeding with infected ticks on noninfected hosts. In some tick species, WNV can be maintained through the transstadial stages of the tick lifecycle, and infected ticks may be capable of infecting hosts through further feeding. When compared to experimental studies with mosquito species (*37*–*39*), ticks are clearly not efficient vectors of WNV and therefore are unlikely to be important vectors for WNV in the current U.S. epidemic. However, our results demonstrate that WNV can persist for a comparatively long time in infected ticks and be transmitted between vertebrate hosts; this finding suggests a reservoir potential of ticks for WNV that justifies further investigation.
